# Airway expression of Transient Receptor Potential (TRP) Vanniloid-1 and Ankyrin-1 channels is not increased in patients with Idiopathic Pulmonary Fibrosis

**DOI:** 10.1371/journal.pone.0187847

**Published:** 2017-11-17

**Authors:** Nicola-xan Hutchinson, Allen Gibbs, Amanda Tonks, Benjamin D. Hope-Gill

**Affiliations:** 1 Department of Respiratory medicine, Princess of Wales Hospital, Bridgend, United Kingdom; 2 Department of Histopathology, Cardiff and Vale University Health Board, Cardiff, United Kingdom; 3 School of Medicine, Cardiff University, Cardiff, United Kingdom; 4 Department of Respiratory Medicine, Cardiff and Vale University Health Board, Cardiff, United Kingdom; National Yang-Ming University, TAIWAN

## Abstract

Dry cough is a common symptom described in patients with Idiopathic Pulmonary Fibrosis (IPF) and impairs quality of life. The exact mechanisms causing cough in IPF remain unclear, however evidence suggests altered cough neurophysiology and sensitisation plays a role; IPF patients have an enhanced cough reflex sensitivity to inhaled capsaicin. The Transient Receptor Potential Vanniloid-1 channel (TRPV-1) has a role in the cough reflex and airway expression is increased in patients with chronic cough. The Ankyrin-1 receptor (TRPA-1) is often co-expressed. It was hypothesised that, like chronic cough patients, IPF patients have increased airway TRP receptor expression. Bronchial biopsies were obtained from 16 patients with IPF, 11 patients with idiopathic chronic cough and 8 controls without cough. All other causes of cough were rigorously excluded. Real-time quantitative Polymerase Chain Reaction was used to detect TRPV-1 and TRPA-1 mRNA expression with Immunohistochemistry demonstrating protein expression. Mean TRPV-1 and TRPA-1 gene expression was higher in IPF patients compared with controls, but the difference did not reach statistical significance. Immunostaining supported these findings. This study suggests that structural up-regulation of central airway TRP receptors is not the key mechanism for cough in IPF patients. It is probable that IPF cough results from altered neuronal sensitivity at multiple levels of the cough pathway.

## Introduction

Idiopathic pulmonary fibrosis (IPF) is a chronic, fibrotic interstitial lung disease of unknown aetiology [1]. Despite new therapeutic strategies the prognosis for the majority of patients remains poor with a median survival of less than 4 years and supportive therapy remains the mainstay of treatment for many [2].

A debilitating dry cough is described in more than 70% of IPF patients [2]. Patients have been shown to have higher 24-hour cough counts compared with controls [3] and this symptom impairs quality of life [4].

The exact mechanisms causing cough in IPF remain unclear. [5]. It is interesting that cough should be such a prominent symptom of a disease that primarily affects the peripheral lung parenchyma as the afferent sensory fibres are concentrated in central airways [6]. There is evidence to suggest that cough in IPF may be a consequence of altered cough neurophysiology and sensitisation. IPF patients have been shown to have an increased cough reflex sensitivity to capsaicin [7, 8].

The capsaicin receptor is a member of the transient receptor potential (TRP) family of ion channels known as transient receptor potential vanniloid-1 (TRPV-1) [10,11]. TRPV-1 is a poly-modal channel which is predominantly expressed in sensory afferent fibres and is activated by capsaicin, noxious stimuli and heat [9, 10]. Immuno-histochemical (IHC) studies comparing bronchial biopsies from patients with and without chronic cough identified a significant increase of TRPV-1 staining nerve profiles, which was positively correlated with capsaicin tussive response [11].

Neurotrophins increase the expression and sensitivity of the TRP channels in the airways [12–14]. There is evidence for increased expression of neurotrophins in the central airways of patients with IPF [8].

Transient Receptor Potential Ankyrin 1 (TRPA-1) is another receptor subtype which is usually co-expressed with TRPV-1 and also responds to noxious stimuli and cold temperatures [15].The evidence for its role in pathological cough is unclear, however given the co-localisation of the two channels and anecdotal evidence that patients with IPF find their cough worse in cold conditions, it is possible that co-operation occurs.

Due to the known concentration of sensory neurones in central airways and the increased levels of neurotrophins in the bronchi in IPF, we postulated that, like chronic cough patients, patients with IPF have increased expression of airway TRP receptors, which could be the basis for increased cough sensitivity in this condition. The aim of this study was therefore to identify whether the expression of TRPV-1 and TRPA-1 was increased in the central airways of patients with IPF.

## Methods and materials

### Subjects

Consecutive patients with IPF were recruited from the Interstitial Lung Diseases Unit at University Hospital Llandough, Cardiff, UK between 2011 and 2014. Three groups of newly referred patients were identified to allow comparison; patients with IPF, idiopathic chronic cough (CC) and ‘normal’ controls (NC).

All IPF patients fulfilled the American Thoracic Society criteria for the diagnosis of IPF [1]. Idiopathic chronic cough patients were defined as those patients with a greater than 3 months history of cough and a normal chest X-ray after the exclusion of other causes of chronic cough as per usual clinical practice [16]. All patients with chronic cough had at least a 1-month trial of treatment with a proton pump inhibitor in order to eliminate gastroesophageal reflux as a cause of cough. ‘Normal’ control patients were identified as patients without cough but were required to undergo bronchoscopy for evaluation of a non-airways centred disorder; such as a pulmonary nodule or single episode of haemoptysis.

Potential confounding causes of cough were identified and all three groups were subject to the same exclusion criteria (Table 1), ensuring all other causes of cough were carefully excluded.

**Table 1:** Study exclusion criteria.

Smoking history within 1 year.
Respiratory tract infection within three months.
Current symptoms of gastroesophageal reflux.
Significant rhinosinusitis symptoms.
Other respiratory diagnosis, including Chronic Obstructive Pulmonary Disease, Asthma*
Other severe systemic illness.
Angiotensin converting enzyme inhibitor therapy

* Co-existent bronchial hyper-reactivity was excluded following thorough clinical assessment, and reversibility testing or methacholine challenge testing were performed where there was clinical uncertainty.

### Protocol

#### Pulmonary function tests

All patients with IPF and idiopathic chronic cough underwent pulmonary function testing [17] as part of routine clinical care. ‘Normal’ control patients underwent spirometric testing at the discretion of the consulting healthcare professional. All ‘Normal’ ex-smokers underwent spirometric testing as a minimum.

#### Assessment of cough severity

All patients with IPF and idiopathic chronic cough were asked to complete a Visual Analogue Score (VAS) and Leicester Cough Questionnaire (LCQ) prior to bronchoscopy [16, 18]. The VAS is a 100 mm linear scale on which patients are asked to mark the severity of their cough. The extremes of the scale were marked 0 (no cough), and 100 (worst cough).

#### Bronchoscopy

Flexible fibre-optic bronchoscopy was performed in all recruited patients according to established guidelines. Bronchial biopsies (maximum 6) were taken using 2.8mm forceps from the segmental carinae of the right lung in each patient. This site was chosen as a consistent landmark within the central airways to enable direct comparison between patients. Biopsies were placed immediately into RNAlater^TM^ (Ambion, Paisley, UK) or buffered formalin. Biopsies stored in RNAlater^TM^ were stored at room temperature overnight and then transferred to -80°C.

#### RNA extraction and cDNA synthesis

Total Ribonucleic Acid (RNA) from the bronchial biopsies was extracted using the acid guanidinium thiocyanate-phenol-chloroform method (TRIzol, Invitrogen, Paisley, UK). Biopsy material was homogenised using a TissueRuptor (Qiagen, Hilden, Germany). RNA concentration was quantified using a ND-1000 spectrophotometer (NanoDrop products, Wilmington, DE, USA.) First strand synthesis was performed using the SuperScript® VILO^TM^ Complementary Deoxyribonucleic Acid (cDNA) Synthesis Kit as per manufactures instructions (Invitrogen, Paisley, UK). Complementary DNA (cDNA) was stored at -20°C.

Quantitative Reverse Transcription Polymerase Chain Reaction (qRT-PCR)The Lightcycler® 2.0 Carousel-based system and Lightcycler® FastStart DNA Master SYBR Green I kit (Roche Diagnostics Ltd, West Sussex, UK) were used to quantify TRPV-1, TRPA-1 and house-keeping genes Hypoxanthine guanine phosphoribosyl transferase (HPRT) and TATA box binding protein (TBP). The qRT-PCR master mix was prepared in a cooling block as per the manufacturer’s protocol. Complementary DNA from bronchial biopsies were diluted 1:2 and cDNA from an A549 cell line was used as a positive control. To obtain mean values, all samples were run in triplicate. A non-template control and Reverse Transcription (RT) negative sample was also used in each run. Genes of interest and primer sequences are outlined in Table 2. Relative change in expression was calculated using qBasePlus software, including corrections for PCR amplification efficiency estimated by a serial dilution series [19]. HPRT and TBP were selected as stably expressed house-keeping genes (Genorm, M value: 0.415, CV: 0.148) and were used for normalisation.

**Table 2:** Genes and their primer sequences.

Name	Accession number	Primer sequence	RNA product size (bp)	DNA product size (bp)	Reference
TRPV-1	NM_080704.3	Fwd: TGGTATTCTCCCTGGCCTTG	188	3763	[20]
		Rv: CTTCCCGTCTTCAATCAGCG			
TRPA-1	NM_007332.2	Fwd: TCACCATGAGCTAGCAGACTATTT	74	1647	[21]
		Rv: GAGAGCGTCCTTCAGAATCG			
HPRT	NM_000194.2	Fwd: TGACACTGGCAAAACAATGCA	94	4893	[22]
		Rv: GGTCCTTTTCACCAGCAAGCT			
TBP	NM_003194.4	Fwd: TCAAACCCAGAATTGTTCTCCTTAT	122	803	[23]
		Rv: CCTGAATCCCTTTAGAATAGGGTAGA			

The gene accession numbers were obtained from the NCBI (National Centre for Biotechnology Information) database. All primers were synthesised by Life Technologies (Paisley, UK). Primers complimentary and specific to the gene of interest were assessed with BLAST. Each primer set was optimised to maximize efficiency. The thermal cycler conditions were as follows: 10 minutes at 95°C, 10 seconds at 95°C, 60 cycles of 5 seconds at 58°C/60°C (TRPV1,TRPA1/TBP,HPRT) respectfully and 5 minutes at 72°C. The melt curve analysis was performed for one cycle, between 65°C and 95°C at a rate of change of 0.1°C/second.

#### Immunohistochemistry

4μm formalin-fixed paraffin embedded cut sections were investigated by immunohistochemistry (IHC) using the DakoFlex™ Envision plus kit (Dako, Denmark) to identify TRPV-1, TRPA-1 and Pan-neuronal Gene Peptide-9.5 (PGP-9.5) a general nerve marker. The manufacturer’s instructions were followed, with primary antibodies added at concentrations: rabbit anti-TRPV-1 1/1000 [24], rabbit anti-TRPA-1 1/600 [21] or rabbit anti-PGP-9.5 1/1600[25]. A negative and positive control was included in each run.

The specimens were evaluated simultaneously by authors NH and AG using an Olympus BX51 microscope. Group identity of the samples were not available prior to the analysis. Antibody staining was recorded as either positive or negative and distribution noted. Any discrepancies in the results were resolved by consensus following reassessment of the sample by both investigators. To quantify any difference in immuno-histochemical staining between the three groups a digital method of quantification was used. Bright-field whole slide images were captured and digitalised using an Axio Scan.Z1 slide scanner with a 20X objective lens and a Hitachi HV-F2025CL camera (Zeiss, Cambridge, UK). The images were analysed using ImageJ version 1.50j [26]. The IHC image analysis toolbox [27] used to detect positively stained colour pixels allowed the area of specific immunostaining for neuronal PGP-9.5, TRPV-1 and TRPA-1 staining to be expressed as a percentage of the total area.

#### Statistical analysis

SPSS version 20 (SPSS Inc. Chicago. Illinois) was used for data analysis. Statistical significance was considered to be <0.05.

The Student t test and Mann-Whitney U test were used to compare parametric and non-parametric continuous data between two groups respectively. One-way Analysis of Variance (ANOVA) and Kruskall-Wallis were used when more than two groups of parametric or non-parametric data were being compared respectively. Chi-squared test or Fishers exact test was used to compare the categorical data, whilst Spearman’s rank correlation was used to assess association between continuous variables.

All qPCR data was Log_10_ transformed to ensure it fitted a normal distribution. Average gene expression between the groups was assessed using ANOVA, however linear logistic regression was also used to account for the other variables within the study.

#### Institutional approval

The study was approved by the Cardiff and Vale University Local Health Board Research and Development Committee and the Local Research Ethics Committee. Subjects provided informed written consent.

## Results

Thirty five patients in total were recruited; 16 patients with IPF, 11 with idiopathic chronic cough and 8 ‘normal’ controls. Demographic, pulmonary function and cough severity data for study subjects are compared in Table 3, with a more detailed demographic profile of the IPF patients shown in Table 4.

**Table 3:** Study subject characteristics.

	CONTROL	IPF	CHRONIC COUGH
Number	8	16	11
Mean age: years	46	70	60
(Min, Max)	(24, 69)	(56, 82)*	(48, 73)^¥^
Gender (M:F)	5:3	14:2	2:9* ^¥^
Mean FEV1: % predicted	94.85	87.21	107.29
(Min, Max)	(84.9, 110.0)ⱡ	(59.7,126.4)	(72.0,145.0)
Mean FVC: % predicted	100.8	82.29	108.65
(Min, Max)	(80.0,133.0) ⱡ	(51.9,113.9)	(78.2,142.3)^¥^
Median TLCO: % predicted		41.15	81^¥^
Median VAS: mm		40	77^¥^
(25^th^, 75^th^ quartile)		(24.8, 62)	(49, 89)
Mean total LCQ score		14.88	10.91^¥^
(Min, Max)		(7.34, 20.21)	(6.75, 13.17)

* p <0.05 compared with control group

¥ p <0.05 compared with IPF group

ⱡ Number = 4

VAS = Visual analogue score; LCQ = Leicester cough questionnaire. A lower LCQ score reflects lower cough-related quality of life.

Definition of abbreviations: F = female; M = male; 95% CI = 95% confidence interval; FEV1 = forced expiratory volume in 1 second; FVC = forced vital capacity; TLCO = gas transfer.

**Table 4:** IPF patient demographics.

Patient	Age	Gender	Smoking status	FVC	TLCO	HRCT
1	75	Male	Non-smoker	51.9	27.4	UIP pattern
2	72	Male	Non-smoker	74.0	33.6	UIP pattern
3	72	Male	Non-smoker	79.5	36.0	UIP pattern
4	56	Male	Ex-smoker	76.0	40.0	UIP pattern
5	74	Male	Ex-smoker	85.8	46.5	UIP pattern
6	71	Male	Ex-smoker	82.8	41.1	UIP pattern
7	67	Female	Ex-smoker	108.3	57.7	UIP pattern
8	74	Male	Non-smoker	90.8	60.6	Atypical for UIP*
9	74	Male	Ex-smoker	84.2	37.7	UIP pattern
10	70	Male	Non-smoker	61.2	63.4	UIP pattern
11	62	Male	Non-smoker	73.2	37.8	UIP pattern
12	65	Male	Non-smoker	77.9	75.7	UIP pattern
13	60	Male	Non-smoker	65.8	41.2	UIP pattern
14	77	Male	Non-smoker	113.9	134.0	UIP pattern
15	69	Female	Ex-smoker	100.7	40.5	UIP pattern
16	82	Male	Ex-smoker	90.6	76.3	UIP pattern

FVC = % predicted forced vital capacity, TLCO = % predicted transfer factor for carbon monoxide, UIP = Usual Interstitial Pneumonia

*UIP pattern confirmed on surgical lung biopsy

As expected patients with IPF had a significantly lower FVC % (p = 0.026) and TLCO % (p = 0.002). Cough was a prominent symptom in all IPF patients studied. Patients with chronic cough had significantly greater cough symptom severity assessed by VAS and LCQ compared to IPF patients (p <0.05).

### TRPV-1 and TRPA-1 receptor gene expression (Fig 1)

The difference in relative expression of TRPV-1 between the IPF group and the control group when gender, smoking status and age were taken into account failed to reach statistical significance (β = 0.137, 95% CI = -0.025, 0.299, p = 0.093). In addition, there was not a statistically significant difference in relative TRPA-1 expression between the IPF group and the control group (β = 0.150, 95% CI = - 0.399, 0.698, p = 0.579), or the CC group and the control group (β = 0.190, 95% CI = -0.338, 0.718, p = 0.466) when gender, ex-smoking status and age were taken into account (R^2^ = 0.065).

**Fig 1:**
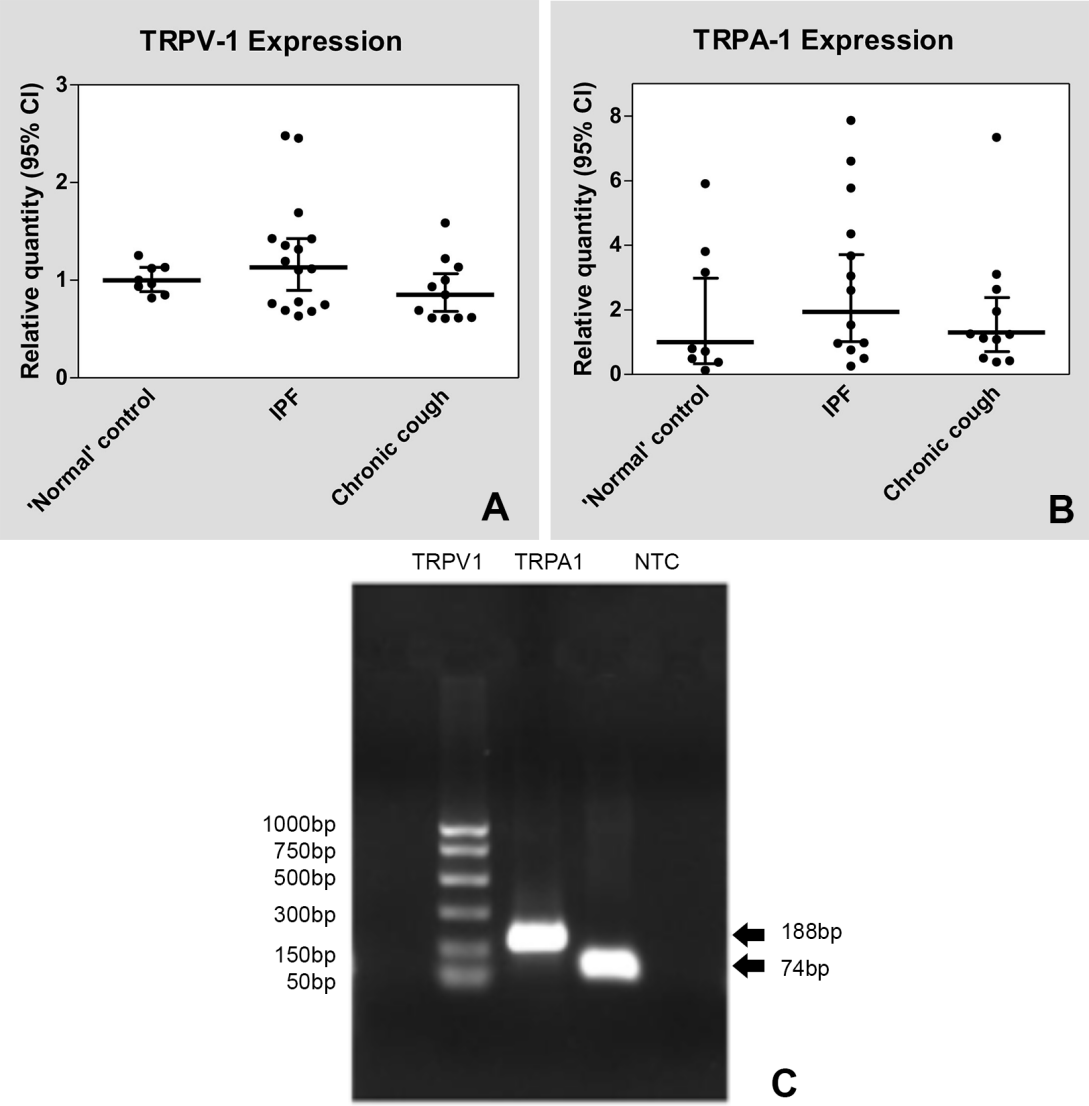
TRP receptor expression. Panel A: Endobronchial TRPV-1 expression. Panel B: Endobronchial TRPA-1 expression. Expression levels of the target genes are shown relative to the cycle quantity (Cq) average. Final expression data is expressed as normalized relative quantity (NRQ) and 95% confidence interval. Panel C: TRPV-1 and TRPA-1 qPCR product gel electrophoresis (1% agarose gel with TBE). NTC = No-template control.

### TRPV-1 and TRPA-1 protein expression

**Fig 2:**
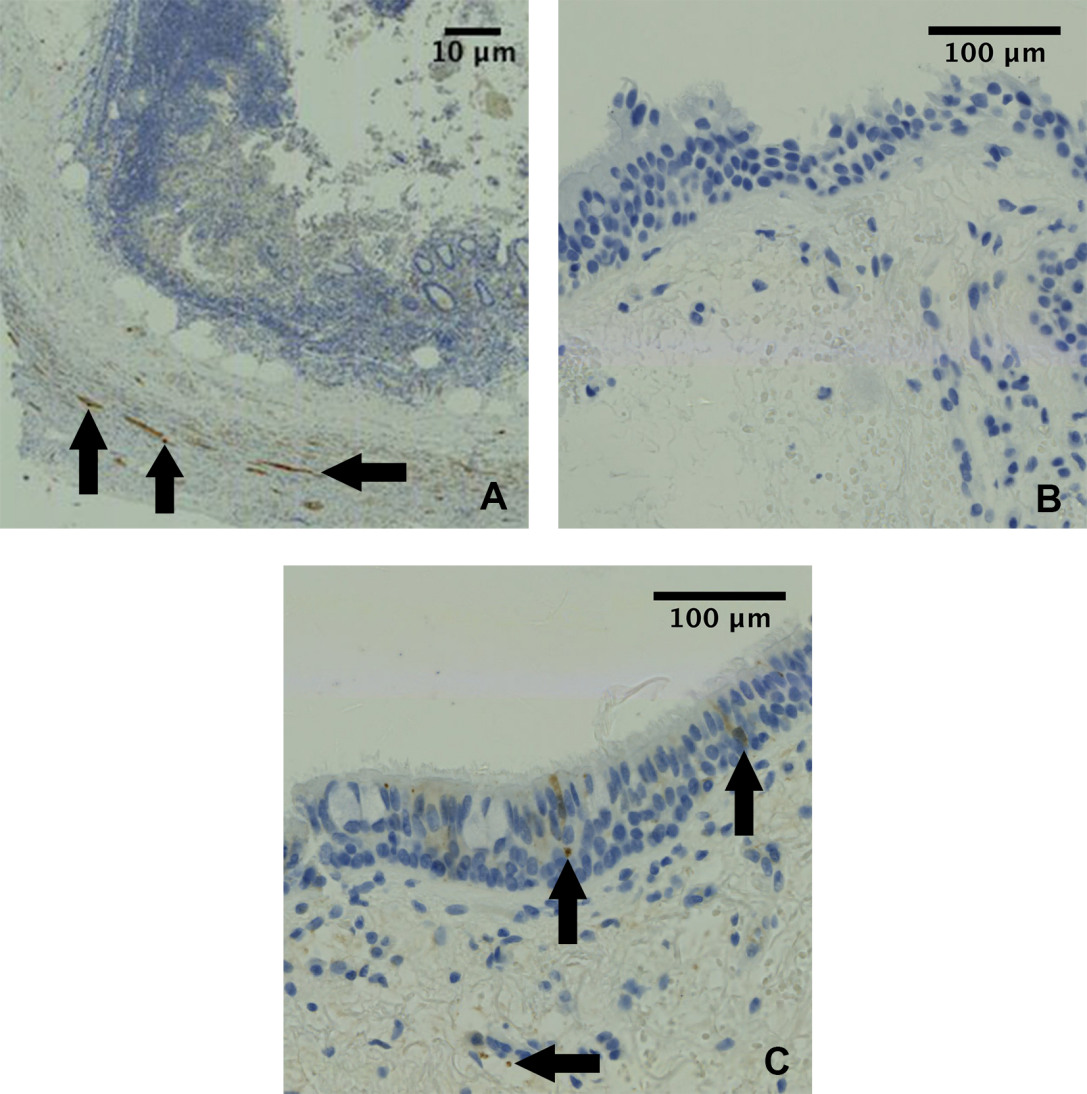
Airway PGP-9.5 Immunohistochemical staining. Panel A: Positive control (appendix tissue), with the black arrows denoting PGP-9.5 specific staining. Panel B: Bronchial sections incubated in the absence of the primary antibody showed no staining (negative control). Panel C: IHC staining of airway nerves in a bronchial biopsy with an anti-PGP-9.5 antibody from a study patient. In agreement with previous studies, neuronal immunostaining for the general nerve marker PGP-9.5 varied among cases and between groups [11, 28]. The median (range) total nerve density of PGP-9.5 in IPF patients was 0.053% (0.002 to 0.29%) and 0.082% (0.009 to 0.63%) in CC patients. There was no statistically significant difference in PGP-9.5 immunostaining between the controls and patients with IPF or CC.

**Fig 3:**
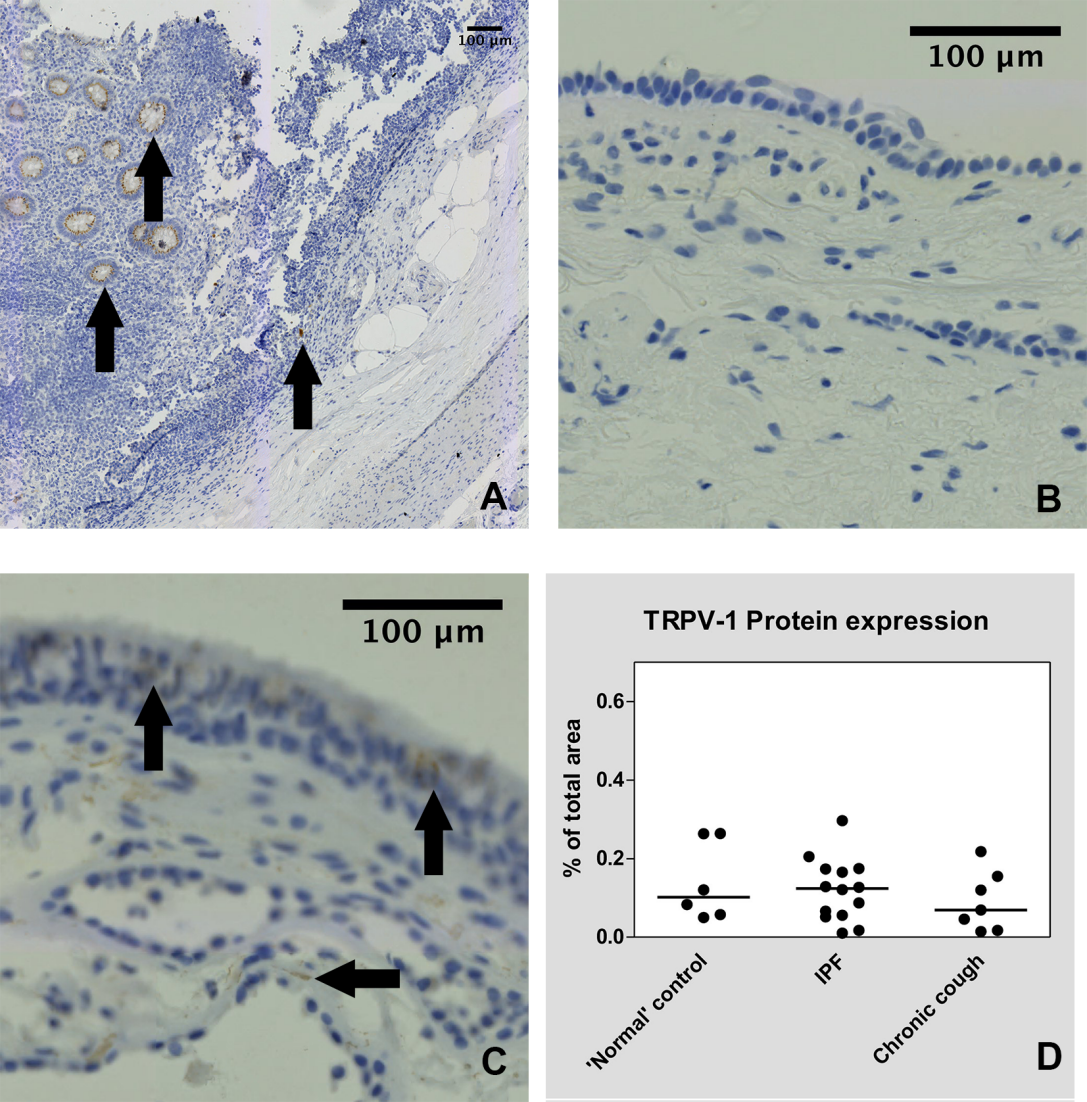
Airway TRPV-1 Immunohistochemical staining. Panel A: Positive control (appendix tissue) with the black arrows denoting TRPV-1 specific staining. Panel B: Bronchial sections incubated in the absence of the primary antibody showed no staining (negative control). Panel C: Positive airway TRPV-1 neuronal staining (vertical arrows) and spindle cell staining in the sub-mucosa (horizontal arrow) from an IPF patient. There was no smooth muscle staining. Panel D: Individual percentages of bronchial airway nerve staining positive for TRPV-1 are shown for each group of patients.

**Fig 4:**
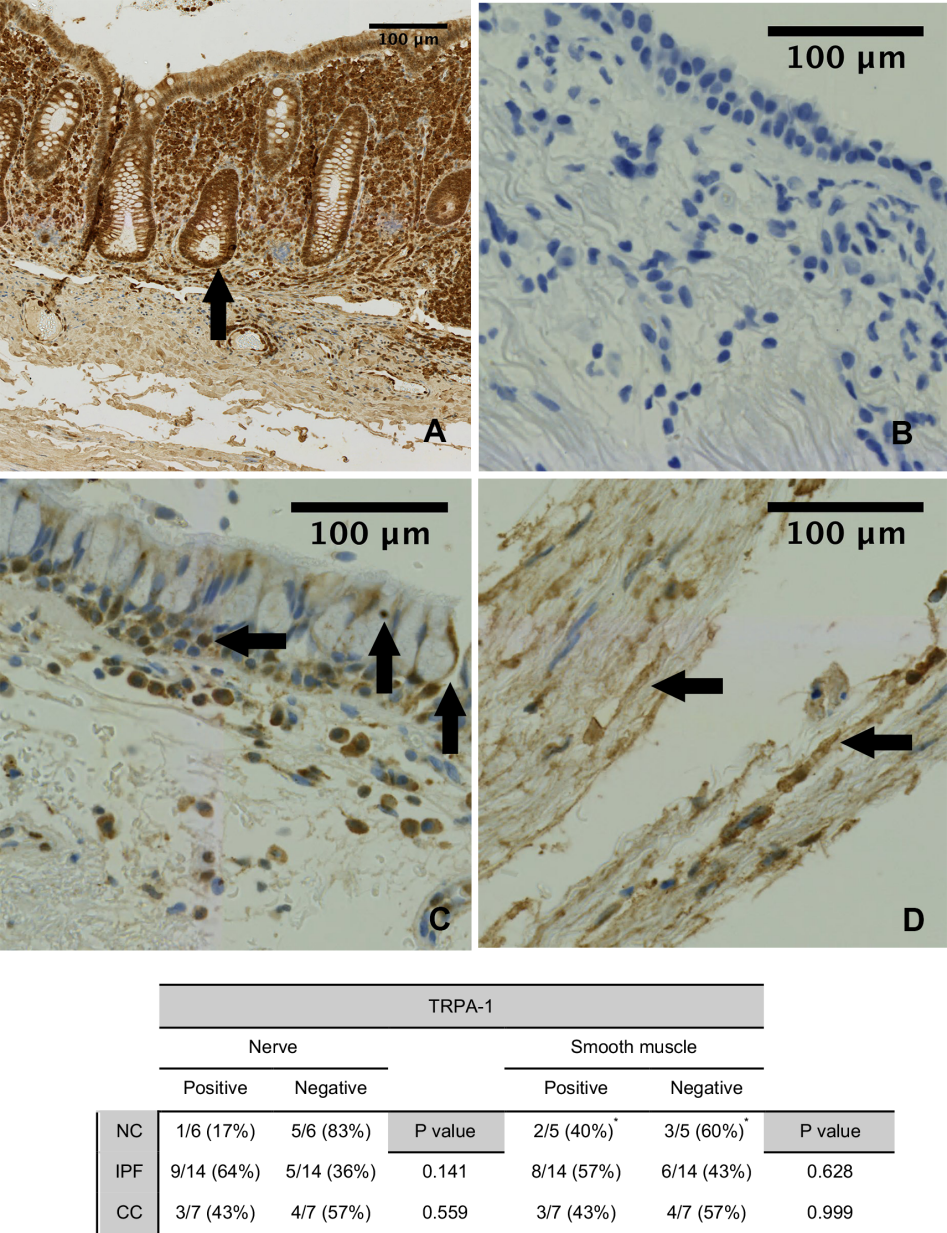
Airway TRPA-1 Immunohistochemical staining. Panel A: Positive control (appendix tissue) with the black arrows denoting TRPA-1 specific staining. Panel B: Bronchial sections incubated in the absence of the primary antibody showed no staining (negative control). Panel C: Positive airway TRPA-1 neuronal staining (vertical arrows) and epithelial cells (horizontal arrow) from an IPF patient. Panel D: TRPA-1 staining within the smooth muscle. Table: Manual assessment of TRPA-1 nerve and smooth muscle staining. There was no statistically significant increase in manual assessment of airway neuronal staining for TRPV-1 or TRPA-1 in the IPF patients compared to other groups.

Immuno-histochemical (IHC) staining was undertaken in 14 IPF, 7 chronic cough and 6 ‘normal’ control patients. Staining the biopsies for PGP-9.5 revealed nerve profile specific staining in the epithelium and sub-epithelium. Figs 2, 3 and 4 show PGP-9.5, TRPV-1 and TRPA-1 staining distribution respectively.

There was evidence of agreement between the manual assessment of IHC staining and the digital quantification.

Median TRPV-1 immunostaining was 0.124% (0.01–0.29%) in IPF patients, which was higher than the median percentage seen in CC and NC patients (Fig 3), however this difference was minimal and did not reach statistical significance (p = 0.53). We also quantified the expression of TRPV-1 in the biopsies as a ratio of the PGP-9.5 expression measured in the adjacent section for each subject. There was no statistically significant difference in TRPV-1 to PGP-9.5 ratio between NC and IPF patients (p = 0.904) or NC and CC patients (p = 0.234).

Quantification of TRPA-1 immunostaining was more difficult as there was epithelial cell and stromal cell specific staining within the smooth muscle as well as neuronal staining. It was not possible to separate the two for analysis, therefore total immunostaining was measured. There was no statistically significant difference in median TRPA-1 immunostaining. In an attempt to assess the differences more accurately a manual semi-quantitative analysis of TRPA-1 expression was performed (data not shown). There was no statistically significant difference between the groups for grade of smooth muscle (p = 0.71) or epithelial (p = 0.18) TRPA-1 staining.

### Association between receptor expression and measures of cough severity

Cough severity as assessed by VAS and LCQ, was not associated with increased airway gene or protein expression of TRPV-1 and TRPA-1 statistically or clinically in terms of minimal important difference (MID) in patients with IPF or CC (data not shown). In addition, there was no association between cough severity and lung function measurements in any group in keeping with previous studies (data not shown) [9].

## Discussion

We have shown that TRPV-1 and TRPA-1 receptor expression is not significantly increased in the central airways of patients with IPF at gene or protein level when compared with patients with chronic cough and normal controls. Cough was the predominant symptom in all IPF and CC patients, however there was no significant association between the level of airway TRP receptor expression and cough severity measured by VAS and LCQ. This study therefore suggests that a structural up-regulation of central airway TRP receptors is not the predominant mechanism for cough in IPF patients.

The rationale for investigating the TRP receptors in IPF is clear as there is evidence for the physiological up-regulation of this pathway; IPF patients have a greater cough reflex sensitivity to inhaled capsaicin [7, 8], and elevated levels of the neurotrophins NGF and BDNF in induced sputum and BAL fluid [5, 8], which are involved in neuronal differentiation and proliferation. Two previous studies have investigated the expression of TRPV-1 in bronchial biopsies of patients with chronic cough, which is one reason why this group was used as a ‘positive’ control. These studies both quantified TRPV-1 expression at protein level.

Mitchell et al. [24] failed to demonstrate a difference in the neuronal expression of TRPV-1, however found a significant difference in the staining of smooth muscle myocytes between CC and control patients, whereas Groneberg et al [11] showed an increase in TRPV-1 positive nerve profiles, with a 4.4-fold increase in the ratio of TRPV-1 to PGP-9.5 staining in CC patients when compared to controls, which was inversely correlated with capsaicin tussive responsiveness. However, they did not demonstrate smooth muscle TRPV-1 staining. The studies therefore report contrasting findings in patients with chronic cough. There are a number of differences between the methodologies of the current study and previous studies, including experimental procedure and patient selection. These differences include variable biopsy site and heterogeneous patient populations in previous studies and are likely to explain the inconsistent findings relating to TRPV-1 expression in patients with chronic cough.

As the focus of our study was on cough in IPF and whether or not aberrant innervation within the central airways is responsible for the enhanced cough reflex particular care was taken to exclude subjects with symptoms of cough of other aetiologies in both chronic cough and IPF patient groups to obtain homogenous patient populations including the exclusion of co-existing emphysema on HRCT scans. This is in contrast to the cohorts of CC patients previously studied which included mixed groups of patients; an important consideration when comparing studies. [11, 24]. Also, to the best of our knowledge, this is the first study to demonstrate both mRNA and protein expression of TRPV-1 and TRPA-1 from airway bronchial biopsies, using previously utilised antibodies and primers with the aim of demonstrating reproducibility.

Although comparable to other similar studies, the sample size is a limiting factor in this study. The comparative group of NC subjects were undergoing diagnostic bronchoscopy therefore were not healthy volunteers. However, as the NC were subject to the same exclusion criteria, this eliminated any confounding effect. The NC group were also not equally balanced with the CC or IPF patients in terms of gender and age. The CC and IPF patients in our study had the expected demographic profiles; IPF predominantly affects males, who have an average age of 74.3 years at the time of diagnosis [29], whereas chronic cough predominantly affects mid-life women [16]. However, the lack of relationship between these factors in a previous study [11] as well as our own would suggest that this did not influence the results.

Our findings show there is not gross up-regulation of TRPV-1 or TRPA-1 in the central airways of patients with IPF. It is important to consider that this study investigated the expression of two members of the TRP receptor family which has 28 members [30]. There is some evidence that other TRP receptors particularly TRPV-4 and transient receptor potential melastatin-8 (TRPM-8) may also play a role [31, 32]. The interactions between these receptors and others such as the acid sensing ion channels (ASICs) are poorly understood and it is likely that a number of receptors and interactions are involved in the cough reflex.

The level at which the airways were sampled is also an important consideration as although the central airways are known to have increased innervation it is possible that there is structural up-regulation of TRP receptors in more distal airways that are directly implicated in the fibrotic process. TRPV-1 receptors have been demonstrated at alveolar level [33], and although the peripheral C-fibres are thought to be inhibitory, there is evidence for increased expression of neurotrophins distally from bronchoalveolar lavage fluid samples in IPF which have been implicated in adaptive responses within sensory neurones, including fibre phenotype switching, and increased expression and functionality of TRP receptors [5, 8].

It is also possible that a physiological up-regulation of TRP receptors through sensitisation results in an increased cough reflex sensitivity rather than a significant structural up-regulation. Inflammatory mediators are known to cause TRP receptor sensitisation therefore small differences in TRPV-1 expression may result in large differences in TRPV-1 sensitivity [12, 34, 35]. Also in support of sensitisation is the mechanism of desensitisation. Desensitisation of the TRPV-1 and TRPA-1 receptors using capsaicin and other agonists has been demonstrated in vitro [36] and has been used widely in the treatment of chronic pain, particularly neuropathic pain [37, 38]. The evidence for inflammation in the central airways, heightened cough reflex sensitivity and the presence of neurotrophins in the sputum of IPF patients also support the notion of sensitisation of TRP receptors in this group. The role of central sensitisation in patients with chronic cough has also recently gained further support and it is possible that central mechanisms are implicated in IPF patients [39]. This is an area that warrants further investigation.

In summary, this study has not shown evidence of an increase in TRPV-1 and TRPA-1 gene and protein expression in the central airways of patients with IPF. This suggests that a structural up-regulation of central airway TRP receptors alone is not the mechanism for cough in IPF patients. However, the clinical observation of heightened cough reflex sensitivity to capsaicin in IPF suggests that an alternative mechanism involving TRP receptors is at work, and we suggest that a complex interplay of several different mechanisms is implicated.

### Ethics approval and consent to participate

The study was registered with the Cardiff and Vale University Local Health Board Research and Development Committee (09/CMC/4620) and the South East Wales Ethics Committee (10/WSE02/62).
